# Evaluation of Neurofeedback Learning in Patients with ADHD: A Systematic Review

**DOI:** 10.1007/s10484-022-09562-2

**Published:** 2022-09-30

**Authors:** Elizaveta Kuznetsova, Antti Veikko Petteri Veilahti, Ruhoollah Akhundzadeh, Stefan Radev, Lilian Konicar, Benjamin Ultan Cowley

**Affiliations:** 1 Faculty of Educational Sciences, University of Helsinki, PO Box 9, 00014 Helsinki, Finland; 2 Department of Communication, Faculty of Humanities, University of Copenhagen Research Unit, Copenhagen, Denmark; 3 Department of Child and Adolescence Psychiatry, Medical University of Vienna, Vienna, Austria; 4 Institute of Psychology, University of Heidelberg, Heidelberg, Germany; 5 Cognitive Science, Department of Digital Humanities, Faulty of Arts, University of Helsinki, Helsinki, Finland

**Keywords:** Neurofeedback, Attention deficit hyperactivity disorder, ADHD, Learning, Learning variability

## Abstract

NFB has a clear potential as a recognised treatment option for ADHD, but suffers from a lack of clarity about its efficacy, still unresolved after multiple controlled trials. Comparing learners and non-learners based on the evolution of patient-level indicators during the trial serves as a ‘natural’ control, and can help elucidate the mechanisms of NFB. We present a systematic review motivated by the need to establish the state of the art of patient learning during NFB treatment in current clinical literature. One particularly striking question we would like to answer here is whether existing NFB papers study learning variability, since only individual performance differences can give us information about mechanisms of learning. The results show that very few clinical trial reports have dealt with the heterogeneity of NFB learning, nor analysed whether NFB efficacy is dependent on NFB learning, even though NFB is believed to be a treatment based on learning to perform. In this systematic review we examine not only what has been reported, but also provide a critical analysis of possible flaws or gaps in existing studies, and discuss why no generalized conclusions about NFB efficacy have yet been made. Future research should focus on finding reliable ways of identifying the performers and studying participants’ individual learning trajectories as it might enhance prognosis and the allocation of clinical resources.

## Introduction

Neurofeedback (NFB) is a type of brain–computer interface, typically using electroencephalography (EEG), in which subjects are presented with information about their electric brain activity in order to consciously control their brain waves. The field of NFB training is not new, but extends to the late 1950s and 1960s (Kamiya, 1962; Sterman et al., 1969), with the first clinical applications specifically on Attention Deficit-/Hyperactivity Disorder (ADHD)-related symptoms in the 1970s (Lubar & Shouse, 1976; Shouse & Lubar, 1979). However, still after 50 years debate continues over the efficacy of NFB for treating ADHD, mostly because existing evidence is mixed and often non-specific, and the mechanisms behind NFB are unclear. One particularly striking question is what role *learning* has to play (Zuberer et al., 2015), since NFB is a *task* that patients must actively perform—not just a passively taken drug. Clinicians often emphasise that patients must learn to perform; however paradoxically, clinical studies do not seem to often report on patient learning.

*In this work, we systematically review how learning in NFB is represented in the clinical literature*, focusing particularly on the reporting of effect variability. This review shows that very few clinical trial reports have dealt with the heterogeneity of NFB learning, nor analysed whether NFB efficacy is dependent on NFB learning, even though NFB is believed to be a treatment based on learning to perform. Thus, we examine not only what has been reported, but also provide a critical analysis of possible flaws or gaps in the conducted studies and try to answer the question why no generalized conclusions about NFB efficacy have been made yet.

Evidence of group effects in the existing body of research, including several randomized-controlled trials (RCTs), shows significant efficacy of standard NFB protocols for both parent- and teacher-rated symptoms (Bakhshayesh et al., 2011; Cortese et al., 2016; Geladé et al., 2018; Gevensleben et al., 2010; Strehl et al., 2017), with a small-to-medium between-group effect size, and large effect size for within-group analysis (Cortese et al., 2016; Van Doren et al., 2019). Significant effects were also sustained at 6–12 months follow-up (Arns et al., 2012; Gani et al., 2008; Gevensleben et al., 2010; Van Doren et al., 2019). NFB training is also not limited to neurologically based difficulties like ADHD and epilepsy (Morales-Quezada et al., 2019; Walker, 2005), but it has been applied to psychogenic disorders like substance abuse disorders (Saxby & Peniston, 1995), psychopathy (Konicar et al., 2015, 2021) and PTSD (Gapen et al., 2016; Walker, 2009). However, the latest sham-controlled RCTs studying ADHD patients have obtained null results. Janssen et al. (2017) demonstrated learning effects in theta and beta frequency bands, but these were not significantly related to symptom improvement in children with ADHD. Their previous behavioral findings (Janssen et al., 2016) also could not confirm the efficacy of theta/beta ratio (TBR) based NFB compared to the control group on parent and teacher reported behavioral outcomes. Latter report by the same group demonstrated significant benefit of NFB compared to the control group at the follow-up but only based on teachers’ report, while there were no differences over time between children who received NFB and semi-active control intervention in parental reports (Geladé et al., 2018). Moreover, some children had different teachers at follow-up. Therefore, this finding should be interpreted with caution. Finally, the most recent paper (Janssen et al., 2020) again revealed no power spectra differences at follow-up between physical activity intervention (semi-active control) and NFB. However, to be noted, the statistical power in all mentioned above papers from Janssen and Gelade research group was reduced due to the inability of the authors to meet pre-registered sample size (Mourik, 2015). Okumura et al. (2019) also failed to prove the efficacy of NFB training implementing slow cortical potential (SCP) based NFB protocol. Even though recent double-blind placebo-controlled RCT (Arnold et al., 2021) showed that both groups significantly improved in parent/teacher-rated inattention from baseline to treatment end and the 13-month follow-up, NFB was not significantly superior to the control condition at either time point on this primary outcome. Thus, the study does not support a specific effect of deliberate TBR based NFB at either treatment end or the 13-month follow-up.

However, the failure to establish the efficiency and specificity of NFB training could reflect the research approach rather than NFB training itself. There is currently no comprehensive understanding of the basic principles of NFB training in any of the many different protocols, which are typically not standardised. The results of NFB training on reducing ADHD core symptoms also depend on the different subtypes of ADHD, and other patient level factors. Recently it was suggested that systematic reviews should consist of at least three RCTs, because the previous requirement of only two independent RCTs might not be enough to overcome publication bias and resolve the mixed findings and implications (Arns et al., 2020). Moreover, relying on symptom outcomes as an efficiency indicator might be considered a limited approach due, for instance, the subpar reliability of teacher ratings (see Minder et al., 2018), though they are widely used alongside parent ratings.

There are several design issues that could affect the existing body of evidence. Some authors have suggested adopting novel outcome measures which allow more reliable assessment of clinical efficacy of NFB treatment; such measures include, the pre-post treatment effect size (ES) both within-group and between-groups, and remission rates (Arns et al., 2020). In addition, studies often consider only the pre- and post-treatment measures rather than data from the entire training period. Data is also undermined by low sample sizes. Most importantly to our systematic review, NFB learning and its relation to the question of efficacy is usually ignored (cf. Zuberer et al., 2015), despite their importance to assessing the underlying mechanisms like the link from self-regulated brain activity across training sessions to behavioral, neuropsychological, and electrophysiological outcomes (Zuberer et al., 2015).

**Fig. 1:**
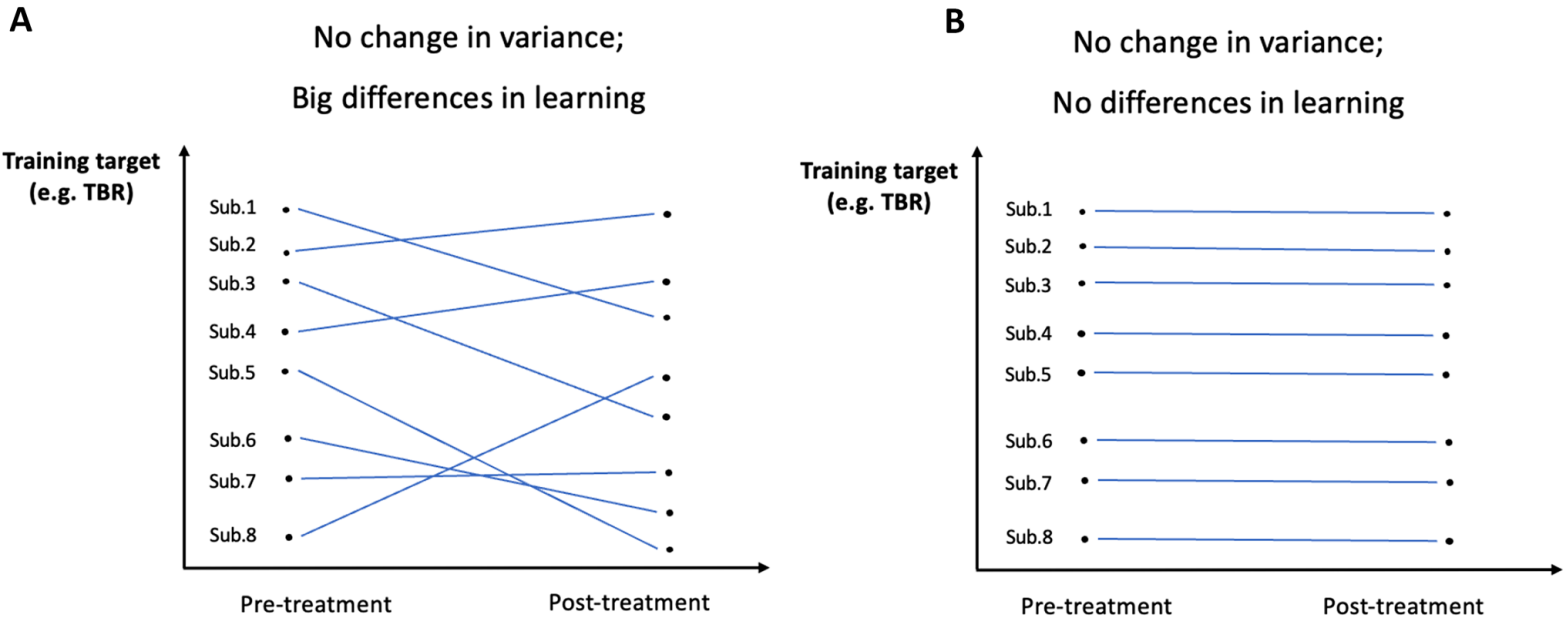
Hypothetical scenarios of subjects’ performance. **a** Represents no change in group variance from pre- to post-treatment, but shows large individual differences in learning performance; **b** represents no change both in group variance and no individual differences in learning performance from pre- to post-treatment

The main focus of our research is whether existing NFB papers study learning variability and what kind of variability they analyse in particular. Hereafter, we define learning as a process that leads to a systematic change in the target of training, keeping in mind that actual operationalisation of learning can vary from study to study (though this would not alter our argument about reporting of subject-wise variance). Both within and between-session learning is of interest as appropriate learning may be seen as developing absolute changes across sessions, or consistency within sessions, or some combination. What remains important is to report those effects of interest on an individual basis. Only individual effect variability can give us information about mechanisms of learning, whereas pooling data from ADHD patients who benefit differently from NFB treatment hides any qualitatively different effects manifested in different subgroups (learners vs non-learners). Mixed results for individual patients may result in the absence of noticeable differences on the group level from NFB pre-treatment to post-treatment, despite some patients significantly improving in performance scores (see Fig. 1 for illustration); in other words, pre- and post-treatment performance variability may equal to zero, despite variation in the patient level effects. With aggregated data, poor performance in terms of the EEG band by some patients could mask a positive learning process among others, which is why reviewing the existing literature on the variability of individual effects is of primary importance. In the discussion part, we consider three main scenarios when reporting the change of variance but not the variance of change, which could lead to wrong conclusions about learning in NFB. We conclude that only papers which study individual effect variability can give us answers to the more specific and interesting questions about the process of learning in NFB.

One such question is what type of learning is involved in NFB, and from what aspects of treatment do effects actually emerge. Traditionally, NFB learning has been viewed as a form of operant conditioning, where the subject learns to regulate the underlying EEG trait in a somewhat automatic fashion in order to normalize the neurophysiological dysfunction they have (Gevensleben et al., 2009). However, more recent papers contrast this ‘conditioning-and-repairing’ hypothesis with the ‘skill-acquisition model’, which requires conscious effort and skill to change the EEG state (Gevensleben et al., 2014; Strehl et al., 2017). This implies that motivational, attributional, and personality factors might play a stronger role (Gevensleben et al., 2014); therefore, learning process outcomes will not occur similarly in all patients in NFB training (Doehnert et al., 2008; Wan et al., 2014). This logically leads to our second and third supplementary questions: is it possible to divide the participants into two groups of learners versus non-learners; and can we predict the learners’ success in self-regulation based on their background characteristics?

On the basis of standardised evidence of learning, we can also examine whether having advanced self-regulation skills leads directly to symptom reduction? Can predictors of learning success equally work as predictors of efficient training? If these questions were answered positively (for tentative evidence, see Veilahti et al. 2021), it would have methodological implications for the study of NFB training efficacy (Gevensleben et al., 2014), making it crucial to identify in advance those who can benefit (e.g. Truant n.d.).

Finally, our last supplementary question focuses on the relation between learning and efficacy of NFB. In particular, we try to find some evidence in reviewed papers on whether good self-regulation skills are crucial in order to obtain symptomatic improvement. Such evidence could cast a new light on the results of prior clinical trials, and profoundly impact the clinical role of NFB. This is crucial as the current, mixed and inconclusive body of research risks leading to disparate policy responses in national healthcare, bearing a direct impact on those afflicted and calling for a more coordinated, fundamental research approach. Some jurisdictions provide (partial) financial support for NFB treatments as a part of clinical-psychological intervention or professional psychotherapy-e.g. Germany, the Netherlands, Austria-whereas other countries, like Finland, have settled on a policy of not supporting NFB treatment.

As a brief overview of these policy differences, the Finnish ADHD Current Care Guidelines for children and adolescents were based on a limited outdated set of meta-analyses of NFB training effects (Cortese et al., 2016; Micoulaud-Franchi et al., 2014; Sonuga-Barke et al., 2013). According to teachers ratings collected in reviewed studies, NFB treatment did not show a significant effect on the core symptoms of ADHD. For adults, Finnish ADHD Guidelines prescribe not to treat ADHD patients with NFB training on the basis of it having an unlikely effect on the treatment of ADHD, though this conclusion is inconsistent with the findings in one major study referred to in the Guidelines. Notably, even though Cowley’s trial (Cowley et al., 2016) indeed did not find evidence for a transfer of learning that was the intended benefit of the intervention, Mayer’s study (Mayer et al., 2016) found significant improvements on all symptom scales with medium to large effect sizes after treatment and six months post treatment. However, due to the fact that no control group data have been reported in this study, ADHD Guidelines assumed that this study can work as evidence against using NFB. Yet the state of literature on NFB efficacy and specificity has dramatically changed since 2016, and therefore the Finnish ADHD Guidelines need to be updated, taking into consideration most recent reviews and meta-analyses (e.g. Arns et al., 2020; Riesco-Matías et al., 2021; Van Doren et al., 2019). There is both a social and economical need for this because ADHD has steadily increased as a reason for disability pension of young adults over the past few decades (Hiilamo et al., 2017). In comparison with the treatment as usual based primarily on pharmacological treatments, the benefits of which seem to require prolonged treatment (Monastra et al., 2002), there is at least tentative evidence that the effects of NFB training could be sustained over a longer term, even after termination of treatment (Mayer et al., 2016).

In contrast, in Austria NFB is considered as a ‘further approach’ in the recommended treatment options for patients with ADHD, alongside relaxation methods such as biofeedback, autogenic training and progressive muscle relaxation (Arrouas et al., 2013). Similarly, the more updated, German Guidelines (Fachgesellschaften, 2017) for the treatment of ADHD, to which Austria mainly refers to, include NFB as a part of interventions on a psychological-psychotherapeutic basis, alongside psychoeducation, psychosocial interventions, and psychotherapy, contrasting with pharmacological approaches. According to the German Guidelines, standard NFB training protocols (TBR, SMR and SCP training), based on learning theories and including transfer exercises, could be implemented within a total duration of 25–30 training sessions for patients older than 6 years as part of a multimodal treatment plan.

Nevertheless, in Austria NFB training is currently neither part of the standard care system, nor reimbursed directly by statutory health insurances. Only in cases of disease-related indications by a physician (according to ICD-10 diagnosis) and doctor’s referral, NFB can be implemented as part of a clinical-psychological intervention or professional psychotherapy. The usual subsidy from the statutory health insurance depends on these conditions. In contrast, in Austria’s private health insurance sector, positive developments could be observed in the last years. More and more supplementary insurances cover all or at least partly the costs of a NFB training, often as stand-alone treatment (up to 80% of the costs could be reimbursed based on referral). This is an obvious sign that the private insurance sector is waking up to considering NFB as a valuable additive or an alternative therapeutic intervention.

Overall, we may observe a positive shift in some European countries towards accepting NFB as an alternative form of treatment for ADHD. However, in other countries the distrust and lack of understanding of the benefits of NFB still exists. A more standardised and comprehensive research approach might motivate health care institutions to reconsider their attitude towards this promising treatment type.

To sum up, the aim of the present review is to establish the state of the art of patient learning during NFB treatment based on the variability of learning which previous debates on the subject largely ignore. In particular, the study (a) conducts a systematic search of the literature to identify studies that assessed learning and learning variability during NFB, and (b) provides a critical analysis of evidence and its possible flaws that need to be addressed in order to make future research on NFB learning and efficacy more standardised and comprehensive.

*Our overarching research question is*: what is the state of the evidence on individual effect variability in the NFB training task, within the literature?

*The supplementary questions are:*

Q1: Do participants show the same level of self-regulation skills or can they be divided into *learners and non-learners*?

Q2: Can we predict success in NFB learning? What are the possible *predictors*?

Q3: How does learning *impact the efficacy* of NFB? In particular, is learning to perform the NFB task a moderator or mediator of efficacy?

Q4: Whether NFB learning is an *operant condition* phenomenon or it works on *skill-acquisition* principles? Or does it imply both of the mechanisms to some extent?

These questions, and our systematic review overall, are exploratory and (given the paucity of known research on the topic) do not aim to comprehensively address the area. We rather aim to illustrate the state of the art, and based on the amount of evidence make some conclusions about how far the state of literature on NFB learning has evolved and what are the possible directions for future research.

## Methods

### Search Strategy

Peer-reviewed journal articles (in English language), published between 1976 and 2021, were searched using electronic databases (Ovid Medline, SCOPUS, EBM Reviews, APA PsycINFO). We conducted two independent searches—one looking for NFB treatment for patients diagnosed with ADHD disorder, and one for other mental disorders attempted to be treated with NFB protocols. We also considered it necessary to conduct a separate search for other mental disorders as NFB efficacy could similarly depend on different types of patients and different protocols, though ADHD remains our main area of interest.

In choosing the keywords for the first search we followed two most recent systematic reviews on ADHD with NFB treatment protocols: Riesco-Matías et al. (2021), Van Doren et al. (2019). The following keyword combinations were used for ADHD search: NFB, EEG Biofeedback, Neurotherapy, EEG feedback, ADHD, Attention Deficit, ADD. The search strategy (databases and search terms) was validated by an experienced research librarian (at the Faculty of Medicine, University of Helsinki).

For the second search the following categories of disorders from the hierarchical Medline search were included: anxiety disorders, disruptive, impulse control, and conduct disorders, dissociative disorders, sleep wake disorders, trauma and stressor related disorders. Scopus database does not have the same hierarchical structure as other three databases and searches can be conducted only by keywords. The keyword combinations used in SCOPUS database were thus different and included particular sub-categories of the disorders mentioned above in order to remain consistent with the Medline search (check supplementary materials for more information). Both ADHD and other disorder searches were limited to randomized control trials.

### Selection Criteria

#### Types of Articles

Primary source of data consists of articles that report cross-sectional or longitudinal associations or experimental results. They were required to have an available full-text copy in order to be included in the review. All secondary sources, including systematic and historical reviews, meta-analyses, comments, expert opinions, recommendations, and replies to comments, were excluded from the study.

#### Exclusion Criteria

The exclusion criteria were as follows: (a) total study sample size less than N = 10; (b) language other than English; (c) paper reports a secondary analysis of a sample already included in the review; (d) study based on biofeedback or passive NFB protocol or protocols under the consideration: sLORETA, Z-score, fMRI, LENS, HEG; (e) presence of severe learning disorders related to IQ, or IQ < 70; (f) more than 25% of participants began or stopped taking medication during the measured learning period. We based our selection on an ordered inspection of the titles and abstracts. If it was not enough to make a decision about exclusion, full-text copies of manuscripts were also reviewed at this stage.

#### Inclusion Criteria

The inclusion criteria for selecting the articles included in our review were as follows:

- any available data on task performance, for two or more time points distributed uniformly with respect to training;
- clinical diagnosis according to best practice for disorder:
  ADHD diagnosed by DSM-III-R, DSM-IV, or DSM-5 guidelines, ICD-10;
  Other disorders: autism diagnosed by ADI and ADOS, other disorders—at least diagnosed by a psychiatrist using replicable methods (e.g. structured clinical interview);
- active (task-based) NFB protocols of multi-sessions:
  TBR (theta-beta ratio)
  SMR (sensorimotor)
  SCP (slow cortical potential)
  alpha

#### Assessment Methodology

To increase the inter-rater reliability (IRR), a third of papers were independently assessed by two reviewers. After comparing the results the IRR was relatively high > 80%. In case of disagreement between the two reviewers concerning a particular paper, consensus was reached by discussion. The rest of the papers were split between those reviewers. At the end of the assessments, four papers were left uncategorized due to some doubts concerning inclusion criteria. Co-author LK was consulted to resolve the issue, since she has the requisite expertise and was not colocated with others (thus unbiased).

## Results

**Fig. 2:**
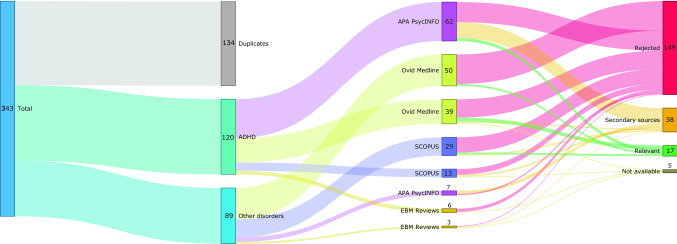
Sankey diagram illustrating the search process of relevant journal articles in four electronic databases

Altogether 343 studies were identified from 4 electronic databases (Fig. 2). After removing duplicates, 209 papers remained for the assessment (120—ADHD, 89—other disorders). Five studies were not available in full-text. After reviewing the full-text of the available studies, only 17 studies met the inclusion criteria for this systematic review: 149 studies were rejected and 38 were secondary source papers. One of the main reasons for exclusion of some full-text articles was the type of the applied NFB protocol. In some studies the term NFB was used interchangeably with other forms of biofeedback, while some other studies used protocols not included in our review. The second most common reason for exclusion was the lack of information on NFB training performance. Third, some articles reported secondary analysis of previously published samples. The categorisation of the studies in this review is presented in Table 1.

**Table 1:** Characteristics of the studies included in this review

Author	N	Target group	Protocol	#	Disorder	Diagnosis
Lubar et al. (1995)	23	Children	TBR	40	ADHD	DSM-III
Heinrich et al. (2004)	22	Children	SCP	25	ADHD	DSM-IV
Bakhshayesh et al. (2011)	35	Children	TBR	30	ADHD**	ICD-10
Bink et al. (2015)	71	Older adolescents	Theta/SMR	37	ADHD	DSM-IV-TR
Janssen et al. (2017)	38	Children	TBR	29	ADHD	DSM-IV
Lee and Jung (2017)	36	Children	Beta/SMR	20	ADHD	DSM-IV TR
Mohagheghi et al. (2017)	60	Children	Theta/beta, Theta/alpha	40	ADHD	DSM-5
Schönenberg et al. (2017)	118	Adults	TBR	30	ADHD	DSM-IV-TR
Strehl et al. (2017)	150	Children	SCP	25	ADHD	DSM-IV-TR
Baumeister et al. (2018)	16	Children	SCP	20	ADHD	DSM-IV
Veilahti et al. (2021)	23	Adults	TBR, SMR	40	ADHD	DSM-IV
Plotkin and Rice (1981)	10	Adults	Alpha	5-7	Anxiety	Welsh A scale of MMPI
Pineda et al. (2008)*	19	Children	QEEG-mu rhythm	30	ASD	ADI-R, ADOS-G
Dadashi et al. (2015)	28	Adults	Alpha, Theta	15+15	Anxiety	DSM-IV-TR
Wang et al. (2016)	14	Adults	Alpha	6	Depression	DSM-V
Schabus et al. (2017)	25	Adults	SMR	12	Insomnia	Edinger et al. (2004)’s research criteria
Konicar et al. (2021)	41	Children	SCP	24	ASD	ADI-R, ADOS-G

Columns are: Author, N = number of participants, Target group, type of NFB Protocol, # = number of sessions, Disorder (which was treated), and Diagnosis (diagnostic classification criteria, e.g. ICD or DSM)

*Study 2

**ADHD w/o hyperactivity or hyperkinetic disorder

Table 1 summarizes the study characteristics, including author, year of publication, number of participants, target group, type of NFB protocol, number of sessions, treated disorder and diagnostic classification. Of the included studies in the review, 11 were investigating the effect of NF treatment on patients with ADHD, two with ASD, two with anxiety, one with insomnia and one with depression. Among those studies concerning ADHD, seven applied the NFB protocol targeting theta frequency band, seven beta band, three SMR, one alpha (in the form of theta/alpha ratio), and three applied the slow cortical potential protocol. The numbers comply with existing literature, considering TBR and SMR protocols the most effective ones for ADHD treatment and most frequently used (Arns et al., 2020; Enriquez-Geppert et al., 2019). Among studies targeting people with other disorders, the most common appeared to be alpha frequency band protocol (3 times), although for ADHD this protocol doesn’t find much support so far (Escolano et al., 2014; Marzbani et al., 2016). The included studies were published between 1981 and 2021, with only two of them published prior to 2000. They were conducted across a number of different countries. Ten out of 17 studies examined children (from 6 to 19 y.o.), six studies examined adults (18–60 y.o.), and one study (Bink et al., 2015) examined both children (< 18 y.o.) and young adults (18–24 y.o.).

Among studies with learning performance data only three (Baumeister et al., 2018; Janssen et al., 2017; Veilahti et al., 2021) made learning the main focus of the paper and studied individual effect variability in the NFB task (see Table 2). For other papers, data from individual performance was pooled together, thus, only group performance variability was analysed. Such papers mostly have their main focus on the efficacy and/or specificity of NFB treatment.

**Table 2:** Learning parameters from studies included in systematic review

Author	Comparable groups	Criteria for learning	Measured variability	Did learning happen?
Lubar et al. (1995)	NFB learners vs NFB non-learners	Theta suppression	Between sessions	In 12 out of 19 patients (63%)
Heinrich et al. (2004)	NFB vs waiting list	Contingent negative variation increase	Pre-training–post training	Yes, on the group level
Bakhshayesh et al. (2011)	NFB vs EMG	Reduced theta/beta ratio	Pre-training–mid-training–post training	Yes, on the group level
Bink et al. (2015)	NFB + TAU (treatment as usual) vs TAU	Theta suppression	First 5 sessions vs last 5 sessions	Yes, on the group level
Janssen et al. (2017)	NFB	Beta enhancement; theta suppression	Within and between sessions	Group level. Between: No—theta, Yes—beta; Within: No—theta, Yes—beta. Individual curves. Between: 39%—theta; 53%—beta; Within: 11%—theta; 82%—beta
Lee and Jung (2017)	NFB + medication vs medication	Theta, SMR, High beta waves, Theta/beta ratio	Pre-training–post training	Left hemisphere: suppressed theta; suppressed high beta. Right hemisphere: suppressed theta; stable beta
Mohagheghi et al. (2017)	Theta/Beta NFB vs Theta/Alpha NFB	Theta Suppression/Beta Enhancement and Theta Suppression/Alpha Enhancement	Pre-training, post training, follow-up	Yes, on the group level
Schönenberg et al. (2017)	NFB vs sham NFB vs meta-cognitive therapy	Reduced theta/beta ratio	Pre-training–mid-training–post training–follow-up	No
Strehl et al. (2017)	NFB vs EMG	Positive slope of correct trials %	Between sessions	In 67.9% of patients
Baumeister et al. (2018)	NFB vs EMG	Positive slope of correct trials %	Between sessions	In eight patients out of 16 (50%)
Veilahti et al. (2021)	NFB TBR vs NFB SMR	Positive slope of time % theta band power below and beta band power above their baseline values	Between sessions	In 11 out of 23 patients (47.8%)
Plotkin and Rice (1981)	NFB Alpha vs NFB Placebo vs waiting list	Alpha suppression for Beta group and alpha enhancement for Alpha group	Pre-training–post training	100% in Beta group, 20% in Alpha group
Pineda et al. (2008)*	NFB mu vs NFB Placebo	Enhanced power in the 10–13 Hz and suppressed power in the 30–60 Hz EMG range	Pre-training–post training	Yes, on the group level
Dadashi et al. (2015)	NFB vs waiting list	Enhanced occipital alpha and theta	Pre-training–post training	Yes, on the group level
Wang et al. (2016)	NFB vs waiting list	Positive difference between alpha power at the right and left frontal lobe	Pre-training–post training	In four out of seven patients (57.14%)
Schabus et al. (2017)	NFB vs sham NFB	Enhanced power in the SMR frequency band	Between and within sessions	No (within sessions); Yes (between sessions)
Konicar et al. (2021)	NFB vs active control (clinical counseling)	Successful SCP differentiation	Between sessions	Yes

Columns are: Author, Comparable groups, Criteria for learning, Measured variability, Did learning happen?

## Discussion

This review has provided an overview of the status of evidence for learning in NFB treatment. Thus far, the state of the art literature on learning in NFB is insufficient for conducting a meaningful meta-analysis or inferring any general conclusions. Among the articles selected for our systematic review only three (Baumeister et al., 2018; Janssen et al., 2017; Veilahti et al., 2021) have main focus on learning and reported individual effect variability in NFB tasks. Lack of studies on patient learning, and unstandardized protocols used in those studies, contribute to the lack of a consensus definition of successful learning because this definition seems unreachable by theoretical means alone. Thus, we ultimately need standardisation of performance requirements, which will help to define the learning itself. There might be several equally valid types of NFB tasks which each have their own definition of learning (e.g. improvement over session baseline, or over a baseline updated every 5 sessions as in the recent iCAN study (Arnold et al., 2021)). Their identification would allow us to operate in terms of “standard kinds” of learning and redefine our research questions to reflect more concrete problems. In the following sections, we discuss the theoretical and methodological implications of the state of the art on NFB learning as reviewed.

### Reporting Individual Effect Variability Data

**Fig. 3:**
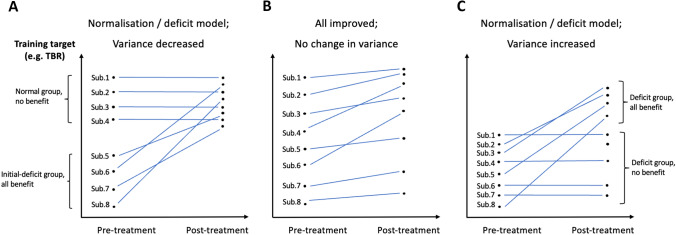
Hypothetical scenarios of subjects’ performance. **a** Shows decrease in group variance due to mitigation of initial large differences between two sub-groups of patients, since one group has a deficit and benefits from the NFB treatment; **b** shows no change in group variance as all patients have improved their learning performance; **c** represents increase in group variance as only some members of the initially low variance group have benefited from the NFB treatment: there is no initial deficit, but learning is still not uniform

In the introduction section, we touched upon the fact that ignoring individual effect variability while reporting only group differences (between pre- and post-treatment variance) can lead to biased interpretations of the results, smoothing out any qualitatively different effects manifested in different subgroups concerning NFB learning. Particularly, assuming that the effect of NFB training is consistently non-negative, we can consider three basic scenarios (Fig. 3).

In scenario A, there are initially large differences between two experimental groups, because some patients have deficits or otherwise negatively differ from the norm. This deficit group might benefit from the treatment irrespective of whether it benefits others (e.g., because NFB would repair a neurological deficit in beta-rhythm activation). Thus, variance on the group level should decrease as the deficit group becomes closer to the norm. This scenario conforms with the ‘conditioning-and-repairing’ model of NFB treatment posited by Gevensleben et al. (2014).

In hypothetical scenario B, everyone’s performance improves, though possibly to different extents. For example, if NFB training works via self-regulation, then the mere act of training might help everyone (following Gevensleben’s skill-acquisition model (Gevensleben et al., 2014), empirically supported by e.g. Veilahti et al. (2021)), regardless of whether the individual does or does not have deficits, analogous to psychostimulants which can benefit ADHD patients, but boost the performance of the non-ADHD population as well. As a result, there will not necessarily be a change in variance, as learning occurs for all the subjects.

The last possible situation (scenario C) is again when some people benefit while others do not, but this time without any initial deficit, thus leading to an overall increase in variance. The difference to scenario 1 is that we assume a specific population with initially low variance, e.g. so-called “low-voltage EEG” phenotype (Johnstone et al., 2005). For this population, learning does not clearly fit either of Gevensleben et al. (2014)’ models, and so does not clarify the treatment mechanism (analogous to selective serotonin reuptake inhibition (SSRI) medication that seems to benefit about 20% when compared with the placebo group based on self-report scores, see Gartlehner et al. (2011).

These hypothetical scenarios can act as a basis of arguing that existing literature is insufficient, i.e, gives us scarce evidence about how learning happens in NFB. They also provide grounds for future research questions concerning learning in NFB.

### Learners vs Non-learners

Doehnert et al. (2008) first proposed the division of the subjects to learners and non-learners. He found that only half of the children trained under the SCP protocol were able to self-regulate. Other more recent papers (Baumeister et al., 2018; Lubar et al., 1995; Okumura et al., 2019; Veilahti et al., 2021; Wan et al., 2014) also found out that approx 50% of participants can be considered learners, regardless of protocol they use. However, in our systematic review Schönenberg et al. (2017) reported that EEG theta/beta-ratio remained unaffected by NFB training in all treatment conditions. In another paper (Bink et al., 2015), learning occurred in the theta frequency band (significant mean difference between first five and last five trials), but not in other frequencies. Coherent with the paradigm shared by this review, the absence of learning could result from inadequate group level analysis, while ignoring individual variability. Indeed, Janssen et al. (2017) reported that on the group level theta remained unchanged over the course of the training, while beta activity increased linearly within training sessions (F(1, 1012.625) = 63.51, p < 0.001) and over the course of the intervention (F(1, 57.461) = 8.60, p = 0.005). However, for individual patients, significant learning curve changes were found for both theta and beta over the course of the intervention. It might be that different directions of individual learning curves in theta band cancel one another on the group level, while the learning process has actually happened for some of the patients, hiding individual learning among some patients.

### Learning as a Skill-Acquisition Phenomenon

Although in many papers NFB learning is still presented as purely an operant-conditioning phenomenon (Bakhshayesh et al., 2011; Janssen et al., 2017; Lee & Jung, 2017; Lubar et al., 1995) and some researchers still employ technique based on the heavily criticised (Pigott et al., 2021) auto-thresholding (Schönenberg et al., 2017), most recent papers provide evidence that NFB is working on skill-acquisition principles (Veilahti et al., 2021) or at least include both of the mechanisms (Arns et al., 2020). In the recent review paper, Arns et al. (2020) claim that intervention might include primary reinforcement of targeted neurophysiological activity via operant conditioning, secondary reinforcement due to the psychological factors implicit in treatment protocols and, in some conditions, synergistic gains when the method is conjoined with other treatments (e.g. psychological therapy, coaching, sleep hygiene etc.). Veilahti et al. (2021), in turn, hypothesized that if NFB learning is not an automatic procedure (as implied in operant conditioning model), there should also be non-neural reasons why some patients fail to learn to self-regulate. Indeed, the results of the paper (Veilahti et al., 2021) show that behavioral scores appeared to have no direct influence from NFB learning, but they are mediated by participants’ scores related to dissociative experiences and the behavioural inhibition system. Moreover, the authors included the inverse trials (use feedback based on the inverse of the standard training target, e.g. regulating theta power up and beta power down) as a pseudo-control condition for both SMR and TBR training protocols and revealed that the effect on behavioral outcomes was different from the one obtained from normal trials. In particular, non-learners’ performance in inverse training didn’t have any effect on behavioral scores such as specific attentional properties like impulse control and variability of response while in normal trials it did. It lets us assume that there should be some other individual factors which influence the success in self-regulation. These include psychological factors like “subjects’ beliefs regarding their ability to gain control over technological devices”, or the lack of suitable mental strategies used in the learning process (Kober et al., 2013). For instance, a large portion of child ADHD patients exaggerate self-efficacy and ability (Owens et al., 2007), whereas low self-esteem seems to make learning slightly more effective (Newark & Stieglitz, 2010). Success also might depend on the chosen sample groups that correlate with specific learning traits.

All of these aspects emphasize the need for more comprehensive data in order to distinguish between the aforementioned scenarios, that is, whether learning occurs among all patients (supports operant-conditioning model) or is specific to enhancing those with particular propensities (in favor of skill-acquisition approach). Overall, there is cumulative evidence that NFB does not work solely on operant-conditioning principles and its cognitively-mediated part should not be neglected. Thus, in future research there is a need to study what makes some people perform and others fail to do so.

### Prediction of Learning Performance

To our best knowledge there are only a few papers which tried to analyse the predictors of learning. For example, in the trial consisting of 25 subjects, Wan et al. (2014) found that the resting alpha frequency before NFB training was a strong predictor of success. Okumura et al. (2019) revealed two pre-training measures that may be possible predictors of success in NF training: score of the Das-Naglieri Cognitive Assessment System Stroop Test (Maekawa et al., 2007) and left prefrontal cortex activation during the Matching Stroop Task. Stroop test reflects the executive function processes (in particular, interference resolution and response inhibition), while PFC plays a central role in performing the task. This result suggests that potential learners may be characterized by relatively good executive function. Veilahti et al. (2021) studied clinically relevant predictive factors. Significant predictive relationships were found in anxiety disorder (GAD), dissociative experience (DES), and behavioural inhibition (BIS) scores obtained during screening. Low DES, but high GAD and BIS, predicted positive learning.

In addition to positive predictors, Alkoby et al. (2018) also ask what allows us to predict the failure of NFB training-not only efficacy, but also inefficacy has to be explained. Factors contributing to inefficacy include psychological factors (e.g. Witte et al., 2013) like “subjects’ beliefs regarding their ability to gain control over technological devices”. Notably, the mental strategies leading to successful learning differ from one protocol to another (Kober et al., 2013). Motivational and mood-related predictors as well as memory and attentional abilities have also been studied (see Alkoby et al., 2018). At the neurological level, a Scheinost et al. (2014)’s pilot fMRI study also investigated differences in brain structure, like the resting state level of connectivity in the anterior prefrontal cortex, as predictors of NFB learning. Thus far, the understanding of which variables can predict successful learning is still in the emerging stage. But prediction of learning can be considered as a question of high importance, as according to patients’ neural capabilities and deficits one can understand who can benefit more, which protocols is better to use, and who should be referred to alternative treatments.

### Link Between Learning and Efficacy

Most of the studies in our review confirmed the efficacy of NFB training (except Janssen et al., 2017) and some of them the superiority of NFB in comparison with other treatment alternatives (Bakhshayesh et al., 2011; Strehl et al., 2017) for behavioral and cognitive outcomes. However, it seems that successful NFB learning does not necessarily mean success in regard to ADHD symptoms (Strehl et al., 2017) and vice versa (those who didn’t succeed in self-regulation might also benefit). Lubar et al. (1995) reported that only some of the outcomes were correlated with learning: learners did better in continuous performance task of attention (TOVA), but did not differ with non-learners on behavioral parental ratings and on IQ measures (both groups have improved). Some researchers did not find any relationship between learning effects and symptoms improvement (Schönenberg et al., 2017) and individual learning curves were not significantly correlated with behavioral changes (Janssen et al., 2017). Baumeister et al. (2018) found similar positive effects of both treatment types and of successful learning for inhibitory control. Finally, Veilahti et al. (2021) assumed that it might even be that the very attempt to self-regulate specific EEG bands is more efficient, in terms of relieving ADHD-related symptoms, than the actual ability to self-regulate (Veilahti et al., 2021), as their study has shown that also non-learners might benefit from NFB training. The same phenomenon was also found for other disorders like anxiety: the study of Plotkin and Rice (1981) demonstrates that significant anxiety reduction can be facilitated by the alpha biofeedback context independent of the effects of alpha training on alpha brain waves themselves. Overall, it seems learning has an unspecific effect on efficacy, and more comprehensive research is needed.

### Limitations of the Review and Future Work

The information provided in the set of relevant papers was far from comprehensive and posed challenges to analysis due to their internal limitations. First, as was mentioned in methods section, we accepted papers with available data on task performance, however, eight studies had their results only in the graphical format: the extraction of numerical data (when possible) has been done manually by the authors of this review and, thus, should be treated as approximation.

Second, different researchers used different measurement units. For frequency bands we found data concerning power, relative power, amplitude, log transformed power, transformed amplitude; for event-related direct-current shifts (SCP protocol) data included amplitude, percentile rank score, the percent of correct answers, and the percent of success rate. Casting the data manually into comparable measurement units might also be a source of distortion. Third, it was mentioned in the introduction section that learning takes place over sessions; however, ability to self-regulate was measured only during pre- and post-intervention in six studies out of 17. In other papers authors measured the training performance at least three times during the treatment.

Fourth, different types of controls were used in the studies. In three (Bakhshayesh et al., 2011; Baumeister et al., 2018; Strehl et al., 2017) semi-active control in the form of EMG have been used; Konicar et al. (2021) used an active control group undergoing conventional treatment, that is, clinical counseling during for patients diagnosed with ASD; Schönenberg et al. (2017), Schabus et al. (2017) and Pineda et al. (2008) conducted sham-controlled studies, Heinrich et al. (2004), Dadashi et al. (2015) and Wang et al. (2016) didn’t use any intervention for the control group (waiting list approach), and five studies didn’t collect any data from the control group, instead comparing the performers vs non performers (Janssen et al., 2017; Lubar et al., 1995; Veilahti et al., 2021) or different types of protocols (Janssen et al., 2017; Mohagheghi et al., 2017; Plotkin & Rice, 1981; Veilahti et al., 2021) within NFB treatment group. Two studies assessed the additive effects of NFB treatment: Lee and Jung (2017) examined the potential effect of NFB for children diagnosed with ADHD beginning a medication trial first and Bink et al. (2015) studied whether NFB is of additional value to treatment as usual (TAU) for adolescents with clinical ADHD symptoms. To separate the effect of NFB from (probably) synergetic effect of NFB + some other type of treatment seems impossible in these two papers.

Last but not the least, one can notice that among those papers which studied learning in more detail, several authors came up with advanced methods. Despite his focus on learning, Baumeister et al. (2018) used quite simple methodology to investigate its effects (learners, non-learners): separate mixed-model analyses of variance (ANOVA). By contrast, Janssen et al. (2017) built the analysis on linear mixed models (LMMs) to study whether children with ADHD were able to learn to adapt EEG theta and beta activity. Authors additionally tested whether a parabolic function would increase the fit of the model. However, sensitivity analyses showed that results remained essentially unchanged when analyses were rerun; thus, quadratic terms did not further improve the linear mixed models. Konicar et al. (2021), on the contrary, assumes that linear models might be misleading and more complex analytic approaches could extract a richer amount of information from the data. Indeed, in Konicar et al. (2021), authors applied a multilevel modeling approach (as it accounts for and quantifies inter-individual variability) in the form of Bayesian treatment and revealed that SCP NFB data suggest a slight preference for a quadratic model compared to a linear model in terms of data fit/complexity trade-off. One issue with quadratic fitting of learning data is that these polynomial regression models represent a family of arbitrary (unbounded) statistical models and thus cannot serve as high-fidelity descriptions of learning. In modelling visuomotor performance, power law curves have often been used to fit the data (Newell, n.d.) because they represent more plausible process models. Consider the contrast of a monotonic power law curve with a concave second order polynomial, which suggests the learned performance can intermittently peak and then get worse again. This does not seem plausible in the visuomotor domain (disregarding noise); however, in NFB it is, indeed, possible that learning can get worse again, e.g., after a prolonged break or due to overtraining. Ultimately, no one yet knows which, if any, single-process model should be used to fit NFB learning data.

Like Konicar et al. (2021), Veilahti et al. (2021) also tackled the problem of individual variability, but focused on temporal variation in the amount of time between sessions. Authors applied a known approach for modeling NFB learning itself (LMM to the logarithm of the training score), but to study temporal variability (which to our best knowledge has never done before) they investigated how individual normal and inverse training trials affected NFB scores over continuous time by means of structural equations modelling with continuous time data (CTSEM). This advanced approach allowed researchers to make more precise conclusions about the processes of learning throughout time. However, that study lacked sufficient sample size to make general insights. Because NFB learning is a complex and heterogeneous process with many aspects like individual variability and continuous timing which should be taken into account, simple linear models may not be the most suitable, or at least should be extended with more sophisticated approaches in order to provide better insight into the underlying processes.

In our systematic review we report only studies on well-defined DSM and ICD diagnoses. We also checked studies in healthy individuals, which we assumed might report individual learning parameters as we have advocated. However, most studies appear to endorse standard two-group comparisons, that is, they do not engage the question of within-group variability and individual differences (Egner & Gruzelier, 2002, 2004; Monastra et al., 2001). Therefore, most papers seem to be designed in a similar way as those concerning clinical population, i.e. where there are some predefined targets while testing the efficiency on these two groups.

There are a few exceptions to this where individual level data is reported (Egner & Gruzelier, 2001; Reiner et al., 2014; Ros et al., 2009; Schabus et al., 2017; Zoefel et al., 2011). In this case, it is usually to demonstrate a correlation between two variables, which again concerns the linking of learning-related variables at the group level, rather than focusing on individual learning differences per se. For example, Egner and Gruzelier (2001) compared SMR and beta1 protocols in twenty-two music students. The behavioral task disclosed a significant reduction in commission errors which were positively associated in regression analysis with learned increases within session in SMR and beta1 amplitudes at the group level. As for neural data, the P300b did not differ for both protocols, each of which disclosed positive correlations with learning indices (SMR, r = 0.49, p < 0.06; beta1, r = 0.55, p < 0.05). Ros et al. (2009), in turn, divided participants into those with the higher and lower improvement in microsurgical technique after the NFB training and retrospectively showed that it was the higher improvement group who showed superior theta/alpha ratio. Reiner et al. (2014) showed that theta training was central to the improvement in speed of performance after sleep by positive correlations between the ratio of theta to beta, a ratio obtained to normalize for individual differences in absolute theta, and the performance gain on all assessments following the first night’s sleep.

Although it would be worth focusing on individual variability in NFB learning also in the non-clinical population in the future, there is an important distinction to be made between clinical and non-clinical studies of NFB learning. That is, the goal of training in non-clinical studies is exactly to learn some type of skill improvement, whereas in clinical studies the goal is symptom relief, to which it is still not known how different types of NFB learning are related. Hence our focus in this review is on clinical studies alone. Future research should focus on reporting individual training results, be standardised, and report training performance measures over the sessions.

## Conclusion

Our systematic review has investigated the state of literature on the question of learning in NFB. Results show that so far the questions of learning in NFB did not get due attention in the scientific community. There is currently no yet standardised approach to study learning and its important aspect-individual effect variability, in particular. What has been done so far seems non-comprehensive and inconclusive. Future research should focus on finding reliable ways of identifying the performers and studying participants’ individual learning trajectories as it might enhance prognosis and the allocation of clinical resources. Increased knowledge of NFB learning not only benefits clinical applications, but could also improve our understanding of underlying mechanisms of NFB, neuroregulation and plasticity.
